# Supplementary material for: The Effect of Leaf Plasticity on the Isolation of Apoplastic Fluid from Leaves of Tartary Buckwheat Plants Grown In Vivo and In Vitro
Source: Plants (Basel). 2023 Nov 30;12(23):4048. doi: 10.3390/plants12234048 (PMC10707844; doi:10.3390/plants12234048)
Supplement: Supplementary file 1 [file plants-12-04048-s001.zip › plants-2691698-supplementary.pdf]

Table S1. Weather conditions during the vegetation period of *Fagopyrum tataricum* outdoor grown plants in 2020-2022 years.

		Humidity		Amount of sunny days			Day time air temperature, °C		
Year		2021	2022	2020	2021	2022	2020	2021	2022
Month	Decade								
May	I	39.7	34.5	7.5	5	5	19	27	14
	II	60.8	49.6	5.5	9.5	1.5	9	26	11
	III	37.4	39.9	8	6	4.5	22	16	23
June	I	30.5	40.5	5	7	5	26	23	25
	II	33.6	37.3	8	7	8	25	32	21
	III	29.6	35.1	4	8.5	6.5	17	30	21
Average (june)		31.2	37.6	5.7	7.5	6.5	22.7	28.3	22.3

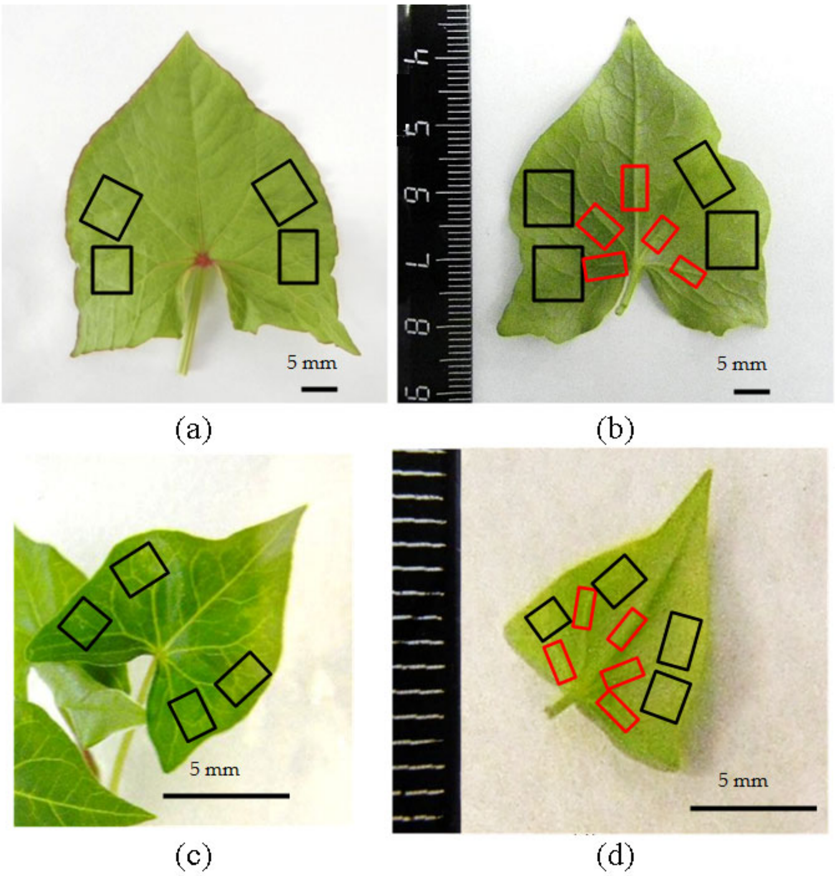

Figure S1. Areas of *in vivo* and *in vitro* leaves, which have been used for morphological analysis and histological and electron microscopy investigations. a – *in vivo* leaf, adaxial surface, b – *in vivo* leaf, abaxial surface, c – *in vitro* leaf, adaxial surface, d – *in vitro* leaf, abaxial surface. Black squares indicate leaf areas, which were used for epidermal strip preparation to study morphology and density of stomatal and epidermal cells, as well as for explant excision for histological and electron microscopy investigations (Figure S1); red squares indicate leaf areas used for preparation of epidermal strips to study CaOx druses morphology and density (Figure S1b, d). CaOx druse study was carried out on preparations of epidermal peels taken off in the areas of first order veins (Figure S1b, d). For studying CaOx druses, the epidermis was taken off only from the abaxial side of the leaf. To obtain a successful preparation for studying, large strips were divided into smaller pieces no more than 2×4 mm. The size of explants for histological and electron microscopic studies was 3×5mm.

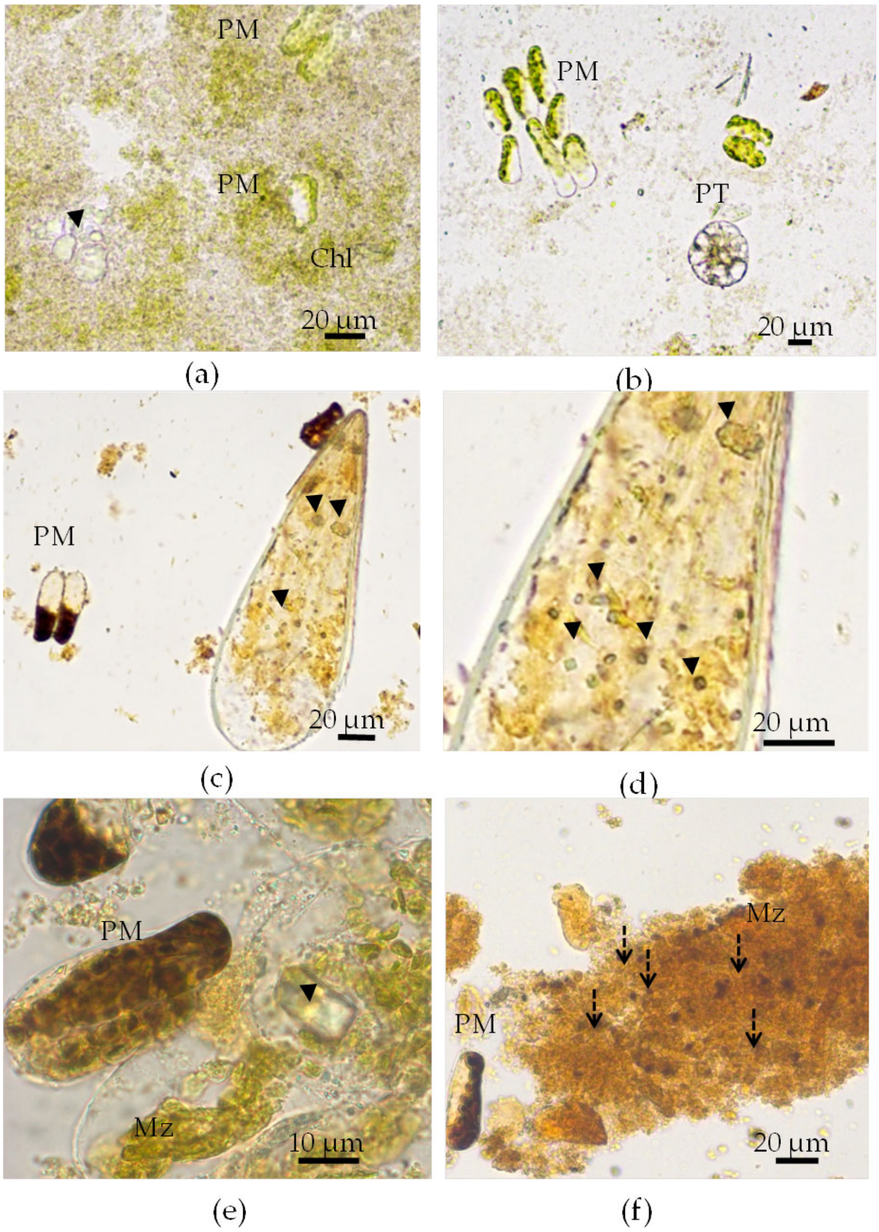

Figure S2. Cytological examination of precipitate after centrifugation at 10,000× g of AWF gathered at 900× g from *in vivo* leaves. a – palisade mesophyll (PM) cells and chlorophyll (Chl) clumps, b – palisade mesophyll and peltate trichomes (PT), c, d – simple trichomes with numerous small CaOx (▼) prismatics, e – idioblasts with one CaOx crystal, f – staining the plaque with Lugol's: randomly scattered starch granules marked with dotted arrow.

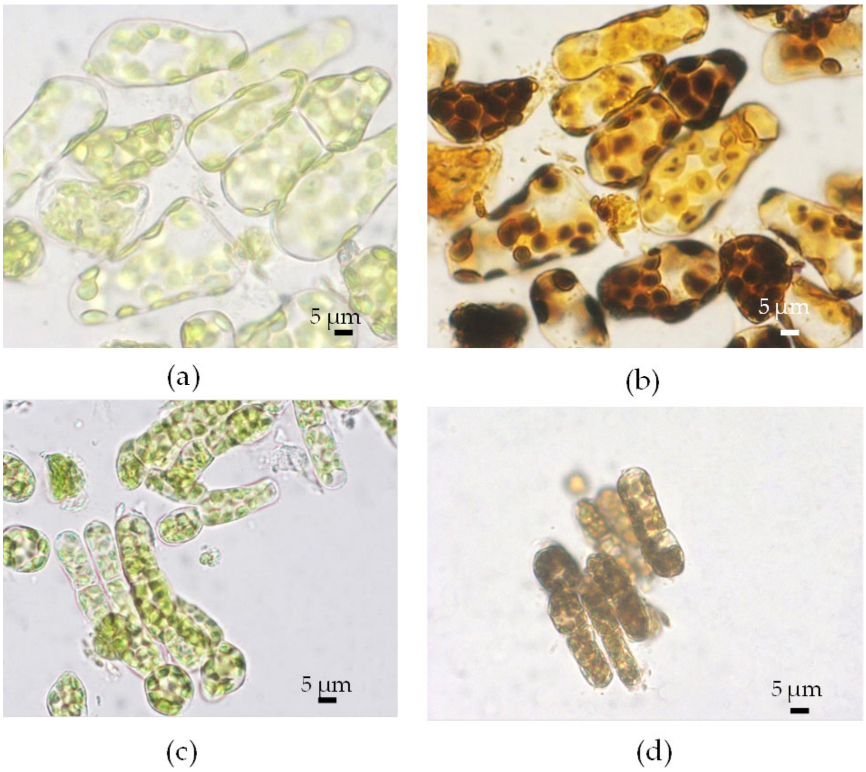

Figure S3. The morphology of palisade mesophyll cells of leaves of buckwheat plants grown in different conditions. a – mesophyll cells of *in vivo* plants, b – mesophyll cells of *in vivo* plants after Lugol's staining; c – mesophyll cells of *in vitro* plants; d – mesophyll cells of *in vitro* plants after Lugol's staining.

Table S2. The size of palisade mesophyll cells of buckwheat leaves grown in different conditions.

**Traits**	**The conditions of plant growth**		
	in vivo	***in vitro***	**PPI**
length, μm	41.43±1.85 *b*	24.13±0.98 *a*	**0.42**
width, μm	13.50±0.46 *b*	7.50±0.18 *a*	**0.44**

Table S3. The coefficients of principal components (PC) according to principal component analysis applied to the epidermal and stomatal characteristics of the adaxial and the abaxial sides of leaves of Tartary buckwheat plants grown *in vivo* and *in vitro*.

**Traits**	**Coefficients of PC1**	**Coefficients of PC2**
Stomatal density	-0,04778	0,62807
Epidermal cell density	-0,40228	0,25534
Stomatal index	0,16293	0,59089
Epidermal cell area	0,3301	-0,20224
Stomatal length	0,37472	0,006
Stomatal width	0,35542	0,28858
Stomatal pore length	0,25529	-0,23759
Stomatal pore width	0,42186	0,04433
Stomatal pore area	0,43647	0,09245

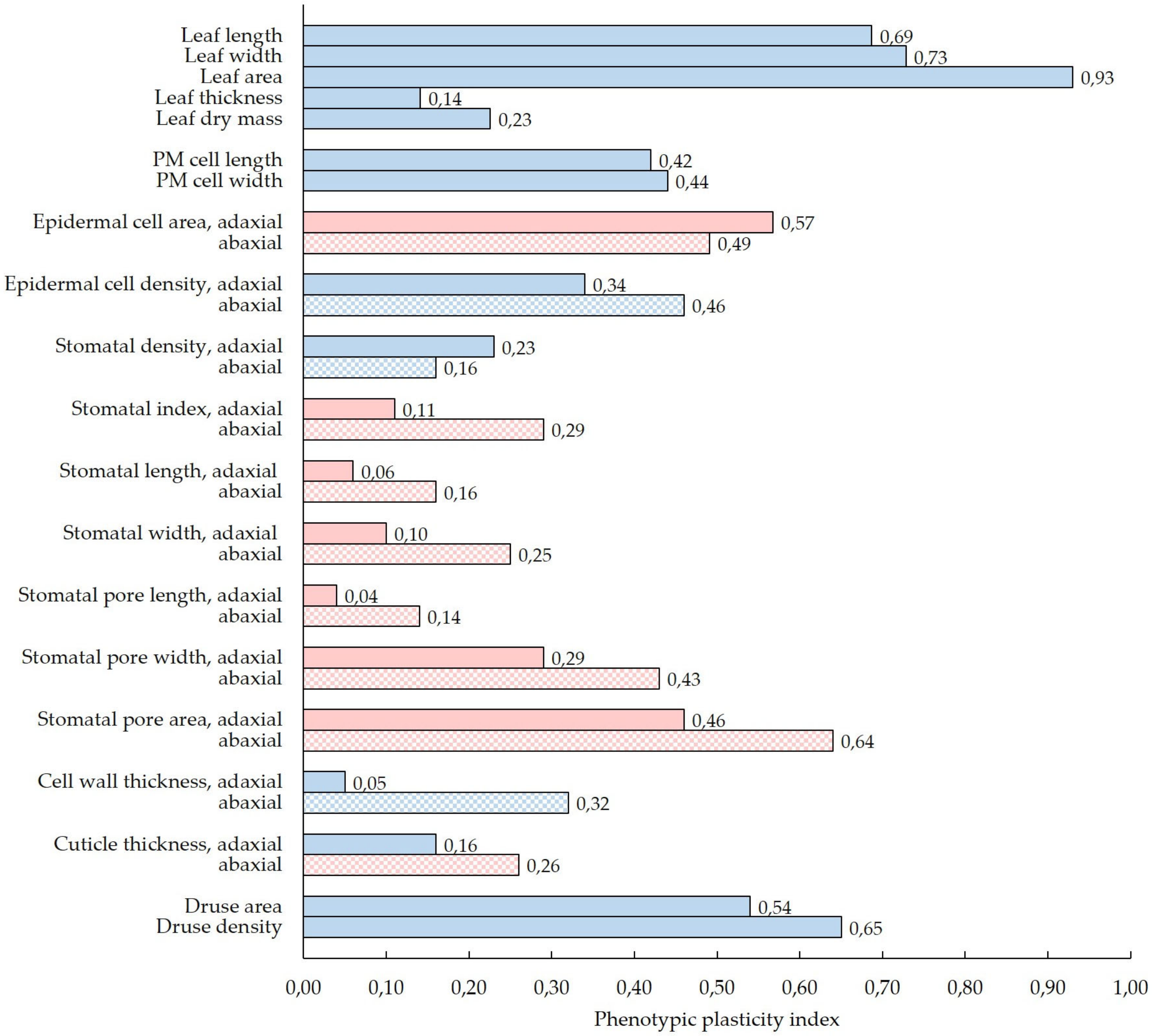

Figure S4. Phenotypic plasticity index (PPI) of *Fagopyrum tataricum* leaves in response to *in vitro* conditions. The color indicates the direction of the trait expression in in vitro conditions: in pink, as increasing, and in blue, as decreasing. If the trait was studied on both sides of the leaf, then the colored column is the PPI for the trait on the adaxial side, and the shaded column is the PPI for the same trait on the abaxial side. PM – palisade mesophyll.

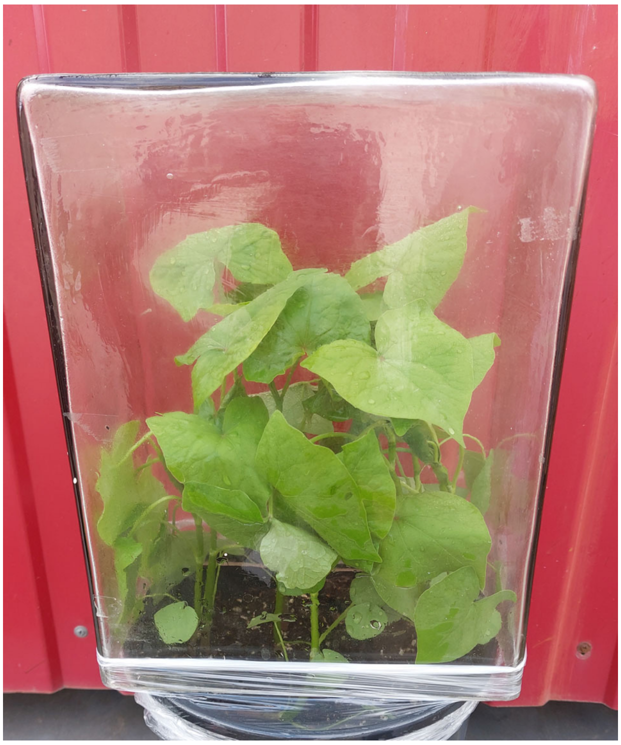

Figure S5. Outdoor Tartary buckwheat plants under “wet chamber” conditions.
